# FFQ-NutriForHer: Reproducibility and Validity of a Semi-Quantitative Food Frequency Questionnaire for Young and Older Women

**DOI:** 10.3390/nu17243811

**Published:** 2025-12-05

**Authors:** Maria Karolina Szmidt, Ewa Sicinska, Olga Januszko, Joanna Kaluza

**Affiliations:** Department of Human Nutrition, Institute of Human Nutrition Sciences, Warsaw University of Life Sciences (WULS-SGGW), Nowoursynowska 166, 02-787 Warsaw, Poland; ewa_sicinska@sggw.edu.pl (E.S.); olga_januszko@sggw.edu.pl (O.J.); joanna_kaluza@sggw.edu.pl (J.K.)

**Keywords:** diet assessment, FFQ, reproducibility, women, validity

## Abstract

**Background**: Despite the growing interest in the relationship of diet and women’s health conditions, a limited number of Food Frequency Questionnaires (FFQs) have been specifically developed and/or validated for use among women, and those that exist have been primarily developed and validated in non-European countries. This study aimed to evaluate the reproducibility and validity of the FFQ-NutriForHer among young and older Polish women. **Methods**: The reproducibility and validity of the 138-item FFQ-NutriForHer were evaluated in 121 young (18–30 years) and 88 older women (70–90 years). Reproducibility was assessed using energy-adjusted Pearson and intraclass correlation coefficients (PCCs, ICCs) from two FFQ administrations. Validity was evaluated by comparing energy-adjusted and de-attenuated PCCs between FFQ-mean and 3-day dietary records. The Bland–Altman method estimated mean differences and limits of agreement. **Results**: The mean PCCs and ICCs for macronutrient reproducibility were 0.69 ± 0.12 and 0.69 ± 0.11 among young women and 0.67 ± 0.06 and 0.65 ± 0.11 among older women, respectively, indicating moderate correlation and reliability in both groups. For micronutrients, the mean PCCs were 0.72 ± 0.05 among young women and 0.69 ± 0.05 among older women, while ICCs were 0.71 ± 0.05 and 0.69 ± 0.09, respectively, indicating a good and moderate correlation and moderate reliability. Mean PCCs for macronutrient validity indicated good concordance with values of 0.51 ± 0.25 among young women and 0.46 ± 0.13 among older women. For micronutrients, the mean PCC for validity was 0.63 ± 0.13 among young women and 0.44 ± 0.14 among older women. Bland–Altman analysis indicated good overall agreement between methods in both groups. **Conclusions**: Given its high reproducibility and satisfactory validity in both age groups, the FFQ-NutriForHer is a reliable tool for assessing dietary intake and exploring its links to women’s health across different ages.

## 1. Introduction

According to the twelfth Council for Responsible Nutrition (CRN)-International conference report, “Women’s health: optimal nutrition through the lifecycle”, sex differences are an important consideration in research, policy development, and optimal health [1]. In recent years, there has been growing interest in studies examining the relationship between diet and women’s health conditions [1], particularly in relation to reproductive health (e.g., dysmenorrhea, endometriosis, menopause) and psychological aspects (e.g., well-being, depression) [2,3,4,5,6,7,8].

At the same time, public health institutions and nutritional scientists are increasingly adopting a lifespan approach, which emphasizes the importance of studying diet across different life stages [9,10,11,12,13]. Observing individuals over the years can provide more comprehensive insights into how long-term dietary habits influence health outcomes. Therefore, there is a recognized need for developing tools that are sensitive to both gender- and age-specific nutritional patterns to more accurately assess the relationship between diet and health conditions [14,15,16,17,18,19].

Food Frequency Questionnaires (FFQs) are a commonly used method to assess diet in studies due to their low cost, ease of administration, and ability to capture habitual intake over extended periods [15,16]. Over the past 40 years, FFQs have been developed and validated in many populations, taking into account cultural and ethnic differences. However, according to a systematic review by Sierra-Ruelas et al. [20], most existing FFQs have been designed for use with both men and women, without specific adaptations to sex. As a result, a limited number of FFQs have been specifically developed and/or validated for use among women, and those that exist are primarily based in non-European countries, such as the United States [21,22,23,24], Japan [25], New Zealand [26], or Mexico [27]. To the best of our knowledge, only a few FFQs have been developed [28] and/or validated [28,29,30] among European women, and none have been specifically designed and/or validated to assess diets across life stages. Most of the FFQs are designed for children, adolescents, or adults [20,21,22,25,26,27,28,29,30,31], limiting their applicability to older populations and restricting the ability to accurately measure generational dietary differences.

Given these limitations, there is a need to develop an FFQ specifically designed for women, which could be used in longitudinal studies spanning from young adulthood to older age. Such a tool would enable the assessment of dietary variations across the lifespan and allow for accurate measurement of the intake of nutrients essential for women’s health, including those relevant to reproductive health and psychological well-being. To address these gaps, the FFQ-NutriForHer was developed as a semi-quantitative tool designed to collect comprehensive dietary data from women across various age groups. This study aimed to evaluate the reproducibility and validity of the FFQ-NutriForHer in two distinct age groups of Polish women: young women (18–30 years) and older women (70–90 years).

## 2. Materials and Methods

### 2.1. Study Design

Recruitment to study was carried out in two age groups of women living in Warsaw, Poland. The young women consisted of students recruited at Warsaw University of Life Sciences who were free of severe diseases (such as cancer, organ failure, diabetes mellitus type I) and food allergy/intolerance, which require a special diet. The older women were recruited from senior clubs, as well as advertisements in the local press. This group consisted of women who lived independently, made their own dietary choices, and, like the younger group, were free of severe diseases (such as cancer, dementia, organ failure, diabetes mellitus type I) and food allergy or intolerance requiring a special diet.

The study was approved by the Ethical Committee of the Warsaw University of Life Sciences (Poland) in 2018 for the young women (No. 25p/2018) and in 2019 for the older women (No. 50p/2019) and conducted in accordance with the Code of Ethics of the World Medical Association (Declaration of Helsinki). All participants signed an informed consent to participate in the study.

**Figure 1:**
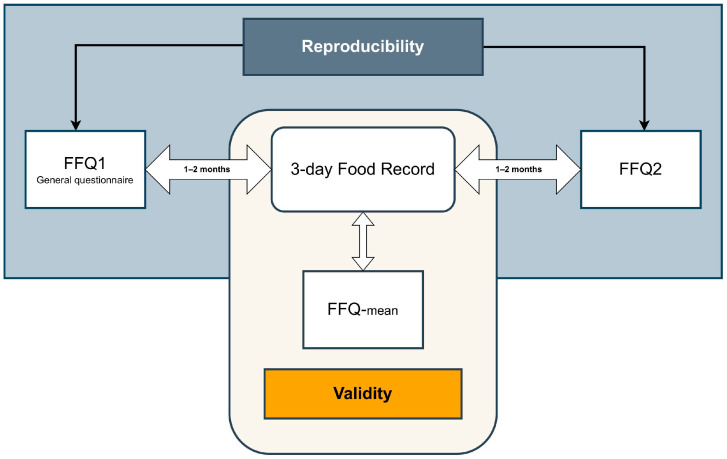
Study design of the FFQ-NutriForHer reproducibility and validation. Abbreviations: FFQ1, food frequency questionnaire 1; FFQ2, food frequency questionnaire 2; FFQ-mean, average values calculated from FFQ1 and FFQ2.

To assess the reproducibility of the data collected using the semi-quantitative paper version of FFQ-NutriForHer, the data were gathered twice with a 2–4-months interval between assessments (Figure 1). To assess validation of the FFQ, a 3-day diet record (3DR) was used. One to two months after completing the first FFQ (FFQ1), the women were asked to record their food consumption over three non-consecutive days. After the next 1–2 months, they were asked to complete the second FFQ (FFQ2). The time intervals were adjusted to mitigate the effect of memory/recall bias. Furthermore, to ensure the independence of responses, the questionnaires were collected sequentially, so participants had no access to their prior responses.

**Figure 2:**
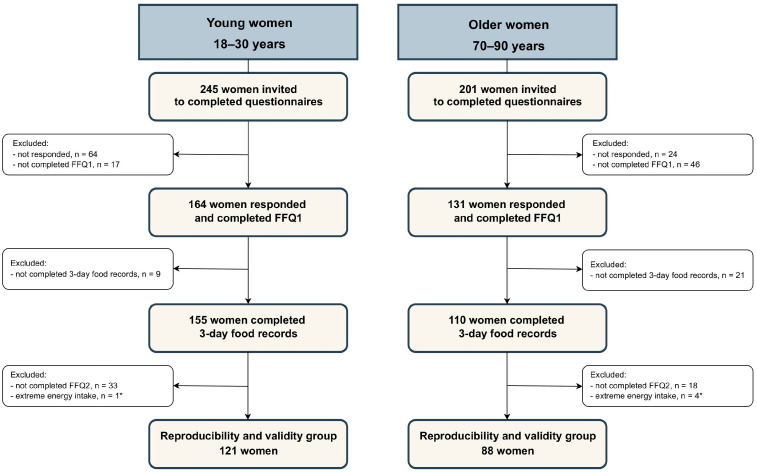
Flow diagram of the women’s recruitment process. Abbreviations: FFQ1, food frequency questionnaire 1; FFQ2, food frequency questionnaire 2. * Energy intake assessed through FFQ1, FFQ2, or 3-day food records was considered implausible if it was <500 or >3500 kcal/day [32].

Out of the 245 young women and 201 older women invited to participate in the study, 81 and 70, respectively, either did not respond or complete the FFQ1—Figure 2. Additionally, 9 young and 21 older women did not record their food consumption using the 3DR method, and 33 young and 18 older women did not complete the FFQ2. Moreover, 1 young and 4 older women were excluded due to implausible energy intake (i.e., <500 or >3500 kcal/day) [32].

### 2.2. General Data

General information such as age, weight, height, place of living (village; town < 100,000 residents; city > 100,000 residents), self-assessed economic status (poor, average, good, very good), smoking status (never, former smokers, current smokers), self-reported health status (poor, average, good, very good), and self-reported physical activity (low, moderate, high, very high) were collected using a general questionnaire at the beginning of the study. Physical activity was self-reported by selecting one predefined category, each accompanied by examples of typical exercises and the corresponding number of hours per week spent on these activities to help respondents choose the most appropriate option. The Body Mass Index (BMI) was calculated by dividing body weight (in kilograms) by the square of height (in meters). The interpretation of BMI values followed the guidelines of the World Health Organization [33].

### 2.3. FFQ Development

The FFQ-NutriForHer was designed and developed to assess food consumption in women across various age groups. It was designed to encompass a wide range of food products that are representative of the dietary habits of European women at different stages of adulthood. This was particularly important, as women’s food choices and dietary habits differ from men’s and tend to change throughout the lifespan [11,34,35,36,37].

A pilot study was conducted among 25 young women and 20 older women to test the clarity of the FFQ and assess whether the range of food items was enough. The results from the pilot study were used to make necessary adjustments to the FFQ. Initially, 135 food products that either comprehensively reflect women’s dietary patterns across the lifespan and/or are known to be important sources of key nutrients such as calcium, iron, fiber, and vitamins were selected [38,39,40]. Following the pilot study, the list was revised. In some subcategories, the number of food items was expanded (e.g., cereal products, dairy products, vegetables), while in others, certain items were combined (e.g., some types of meat, cream, and beer). As a result of these adjustments, the developed FFQ-NutriForHer includes 138 food items. Another objective in developing the FFQ-NutriForHer was to ensure its ease of completion across various age groups of women. To achieve this, the questionnaire was designed with clear instructions on the first page, accompanied by an example, to ensure proper completion. Furthermore, the font size, checkboxes for selecting frequency, and the space allocated for providing an average size of 1 portion were adjusted to be optimal for women in various age groups.

### 2.4. Dietary Assessment

#### 2.4.1. Food Frequency Questionnaire (FFQ)

The semi-quantitative FFQ-NutriForHer includes 138 food items grouped into the following categories: cereal products (n = 12); milk products (n = 12); meat and poultry (n = 10); eggs, fish and seafood (n = 9); vegetables (n = 21); fruits (n = 14); legumes (n = 5); nuts and seeds (n = 11); sugar and confectionery (n = 13); non-alcoholic beverages (n = 9); alcoholic beverages (n = 5); fats and spreads (n = 11); other products (n = 6). Additionally, the questionnaire contained seven extra blank fields that allowed women to specify the consumption frequency of products not listed within selected groups (other fish, other seafood, other vegetables, other juices, other alcoholic beverages, other nuts, and other fats). The FFQ-NutriForHer is presented in the online Supplementary Materials (Supplementary S1—English version; Supplementary S2—Polish version).

The women were asked to indicate the average frequency of consumption for the food items listed in the FFQ over the past year by selecting one of eight predefined categories: less than once a month; 1–3 times per month; 1–2 times per week; 3–4 times per week; 5–6 times per week; once daily; twice daily; or three times daily or more. Additionally, the women were asked to record the average amount or portion size of each item consumed. Portion sizes were documented in grams, milliliters, or household measures such as pieces, teaspoons, or slices. The reported consumption frequency was converted to a daily frequency and multiplied by the corresponding portion size to calculate the daily intake in grams.

#### 2.4.2. 3-Day Food Record (3DR)

Women were asked to complete diet records for three non-consecutive days (including one weekend day), documenting all consumed food products, dishes, and beverages. Portion sizes were specified using household measures or weight units (grams and/or milliliters). A detailed instruction manual and an example of proper completion were provided alongside the food diary. Additionally, participants were asked to include recipes for all home-prepared foods and labels for store-brand products. All 3DRs were verified for accuracy and completeness by qualified nutritionists.

The energy and nutritional value of the diet were calculated using the electronic version of the Tables of Food Composition and Nutritional Value [41] and were adjusted for technological losses. All micronutrient intake was derived from food, including obligatory fortification, while supplement use was not considered.

### 2.5. Statistical Analysis

All analyses were performed in two separate groups: young women and older women. Descriptive statistical analysis was performed for demographic characteristics, presenting the results as numerical and percentage distributions. The relationships between qualitative variables between young and older women were analyzed using the Chi-square test. *p*-values ≤ 0.05 were considered statistically significant.

Based on data collected from FFQ1, FFQ2, and 3DR, as well as for FFQ-mean (mean intake of FFQ1 and FFQ2), energy and nutrient intakes were presented using medians with 25th and 75th percentile values. Before analyses, all dietary data were log-transformed to achieve normal distributions. Additionally, using the residual method, energy-adjusted nutrient intake was calculated separately for young and older women based on the mean energy intake assessed using the 3DR method (1642 kcal/day for young and 1614 kcal/day for older women) [42].

#### 2.5.1. Reproducibility of the FFQ-NutriForHer

To evaluate the reproducibility between FFQ1 and FFQ2, two coefficients were used for both crude and energy-adjusted values: (1) Pearson correlation coefficients (PCCs), and (2) intraclass-correlation coefficients (ICCs) [43]. PCC values ranging from 0.50 to 0.69 were interpreted as moderate correlation, and those ranging from 0.70 to 0.90 indicated good correlation [44]. ICC values were interpreted as follows: below 0.50 indicated poor reliability, 0.50–0.74 moderate reliability, 0.75–0.90 good reliability, and any value above 0.9 indicated excellent reliability [45].

#### 2.5.2. Validity of the FFQ-NutriForHer

To evaluate the relative validity between data obtained from the two FFQs (FFQ-mean) compared to those collected using the 3DR method, Pearson correlation coefficients were calculated for both crude and energy-adjusted nutrient values. Additionally, because day-to-day within-person variance tends to attenuate the correlation between FFQ and diet records, Pearson correlation coefficients were calculated after de-attenuating crude variables as well as energy-adjusted variables. For de-attenuation, the Beaton et al. [46] correction was used: $r_{v}=r_{0}\sqrt{1+\frac{\lambda }{n}}$ where r_v_ is the true correlation, r_o_ is the observed correlation between the FFQ-mean and average of the 3DR, λ is the within-person and between-person variance ratio in nutrient intake derived from the 3DRs, and *n* is the number of replicates (n = 3). Considering that dietary assessment data are generally characterized by inherent variability and measurement errors, we interpreted validity using the cut-off points proposed in the framework by Willett and Lenart [47]. Accordingly, adjusted and de-attenuated PCCs between 0.45 and 0.7 were considered indicative of good concordance between the two methods.

Moreover, women were classified into quartiles of nutrient intake to evaluate the ability of the FFQ-NutriForHer to categorize an individual’s nutrient intake similarly to the 3DR. Agreement between nutrient intake from FFQ-mean and 3DR was assessed by determining whether FFQ-NutriForHer classified individuals into the same or adjacent quartiles (±1 quartile) as the 3DR. Gross misclassification was defined as assignment to opposite quartiles (Q1 vs. Q4 or Q4 vs. Q1).

Furthermore, agreement between nutrient intakes obtained from FFQ-mean and 3DR was also assessed using the Bland–Altman method [48]. This method involves calculating the mean difference between the two methods, determining the limits of agreement (LOA), and illustrating the results as plots. For each participant, the difference between the two methods (y-axis) is plotted against their mean (x-axis), allowing visual assessment of potential systematic bias, specifically whether one method consistently overestimates or underestimates intake compared to the other. The mean difference between the FFQ-mean and 3-DR represents the average bias, while the LOA (mean difference +/− 1.96 standard deviations) indicates the range within which most individual differences are expected to fall. Narrower LOA suggest better agreement between the methods. Figures illustrating the Bland–Altman plots for nutrients important for women in different age groups were provided.

The sample size for Pearson’s correlation analysis was determined using Fisher’s z-transformation. Based on the expected correlation coefficients [47], and assuming a two-sided significance level (α = 0.05) with 80% statistical power, the required sample sizes ranged from 14 participants (for PCC = 0.70) to 47 participants (for PCC = 0.45). To account for an anticipated 10% dropout rate, the target sample sizes were increased to 16 and 53 women, respectively.

All statistical analysis was performed by using STATA 16.1 software (StataCorp., College Station, TX, USA).

### 2.6. Quality of Validation

To assess the quality of the validation, Step 1 of the EURRECA (acronym of EURopean micronutrient RECommendations Aligned Network of Excellence) tool was used [49]. This tool was specifically designed to evaluate the quality of dietary intake validation studies. The scoring system ranges from 0 to 7 points and consists of five components: sample size and heterogeneity (max. 1 point), statistical assessment (max. 3 points), data collection procedure (max. 1 point), consideration of seasonality (max. 0.5 points), and supplement use (max. 1.5 points). Based on the final score, validation studies are classified as follows: poor (<2.5 points), acceptable/reasonable (2.5 to <3.5 points), good (3.5 to <5.0 points), or very good (≥5.0 points).

## 3. Results

### 3.1. Study Population

A total of 209 women participated in the study, including 121 young women and 88 older women. The mean age of the young women was 22.6 ± 1.6 years (median 23.0), while the mean age of the older women was 74.7 ± 5.1 years (median 73.0). Most young women had a normal body weight, lived in large cities, and had at least a good economic status (Table 1). Most of them were non-smokers, self-reported good or very good health, and had moderate physical activity. In contrast, less than half of the older women had a normal body weight, a good or very good economic status, and had never smoked. Most older women lived in large cities and had a very low or low level of physical activity. Significant differences were found between the two groups for all characteristics except place of living.

### 3.2. Reproducibility

For both young and older women, median energy assessed with FFQ1 was slightly higher than with FFQ2 (1650 vs. 1567 and 1627 vs. 1497 kcal/day, respectively)—Table 2. The PCC for energy reproducibility was 0.71 among young women and 0.67 among older women, indicating good and moderate correlation, respectively. The mean Energy-adjusted PCCs for macronutrient reproducibility between FFQ1 and FFQ2 were 0.69 ± 0.12 among young women and 0.67 ± 0.06 among older women, indicating a moderate correlation in both groups. For young women, the Energy-adjusted PCCs for macronutrients ranged from 0.55 (carbohydrates) to 0.84 (cholesterol), while among older women ranged from 0.59 (fat) to 0.74 (dietary fiber). The mean Energy-adjusted PCCs for micronutrients were 0.72 ± 0.05 among young women and 0.69 ± 0.05 among older women, indicating good and moderate correlation between the two FFQs, respectively (Table 3). The Energy-adjusted PCC values for the reproducibility of FFQ1 and FFQ2 for micronutrients ranged from 0.62 (vitamin C) to 0.78 (cobalamin and magnesium) among young women and from 0.59 (vitamin A and vitamin D) to 0.78 (magnesium) among older women.

The ICC for energy intake between FFQ1 and FFQ2 was 0.68 among young women and 0.66 among older women, indicating moderate reliability in both groups. The mean Energy-adjusted ICCs for macronutrients were 0.69 ± 0.11 among young women and 0.65 ± 0.11 among older women, showing moderate reliability in both groups—Table 2. The Energy-adjusted ICC values for macronutrients ranged from 0.57 (fat) to 0.84 (cholesterol) among young women and from 0.51 (fat) to 0.78 (dietary fiber) among older women. Similarly, in both age groups, the mean Energy-adjusted ICCs for micronutrients indicated moderate reliability and were 0.71 ± 0.05 among young women and 0.69 ± 0.09 among older women—Table 3. The Energy-adjusted ICC values for micronutrients ranged from 0.63 (vitamin C) to 0.81 (magnesium) among young women and from 0.48 (vitamin A) to 0.84 (magnesium) among the older women.

### 3.3. Validity

The mean Energy-adjusted & De-attenuated PCCs for macronutrients indicated good concordance between FFQ-mean and 3DRs, with average values of 0.51 ± 0.25 among young women and 0.46 ± 0.13 among older women—Table 4. The Energy-adjusted & De-attenuated PCCs ranged from 0.19 (carbohydrates) to 0.82 (cholesterol) among young women and from 0.34 (protein) to 0.67 (cholesterol) among older women. The mean Energy-adjusted & De-attenuated PCCs for micronutrients were 0.63 ± 0.13 among young women and 0.44 ± 0.14 among older women (Table 5). For young women, the Energy-adjusted & De-attenuated PCCs ranged from 0.43 (pyridoxine) to 0.86 (vitamin D), while for older women, they ranged from 0.24 (thiamine) to 0.77 (vitamin D). Aside from vitamin D, the highest Energy-adjusted & De-attenuated PCCs were observed for cobalamin (0.84), iron, and riboflavin (0.71) among young women, and for vitamin C (0.54), calcium (0.53), and riboflavin (0.51) among older women.

The agreement between methods, measured as nutrient intake classified into the same or an adjacent quartile, was high. Among the young women, it was 81.3 ± 3.1% for macronutrients and 81.2 ± 3.5% for micronutrients, while among older women, it was 76.4 ± 4.4% and 73.4 ± 3.6%, respectively (Table 4 and Table 5).

**Figure 3:**
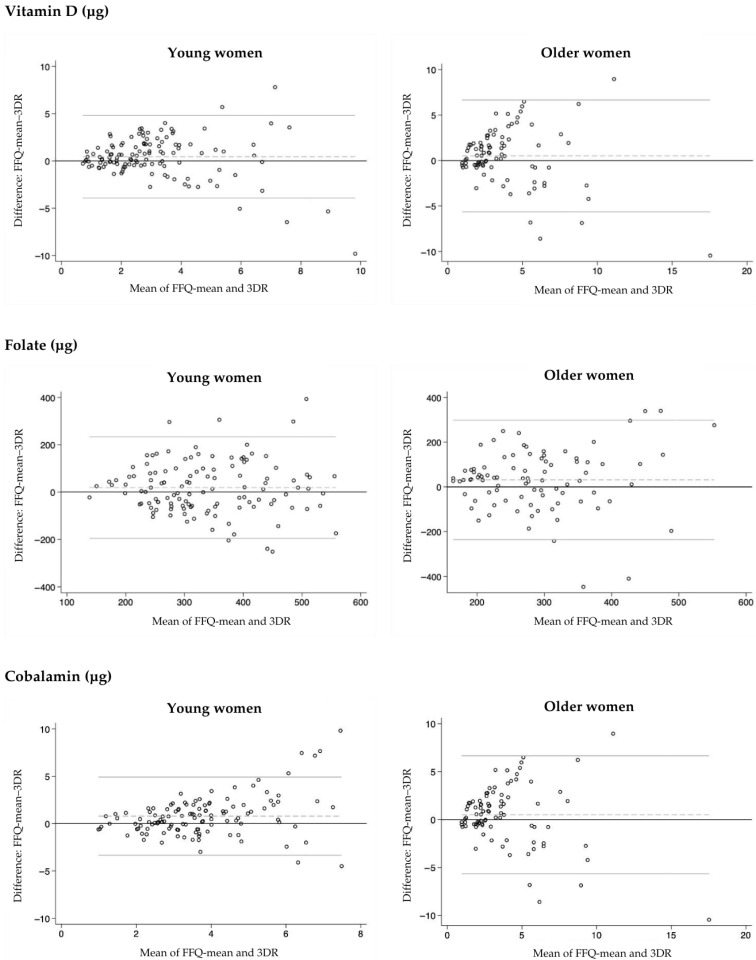
Bland–Altman plots for vitamin D, folate, and cobalamin in young and older women. The plots display the agreement between the FFQ-mean and 3-day diet record (3DR) method for selected vitamins. Each scatter plot is constructed with the mean of FFQ-mean and 3DR on the x-axis and the difference between FFQ-mean and 3DR on y-axis. The dashed line represents the mean difference in intake between the two methods, while the solid thin lines indicate the 95% limits of agreement.

**Figure 4:**
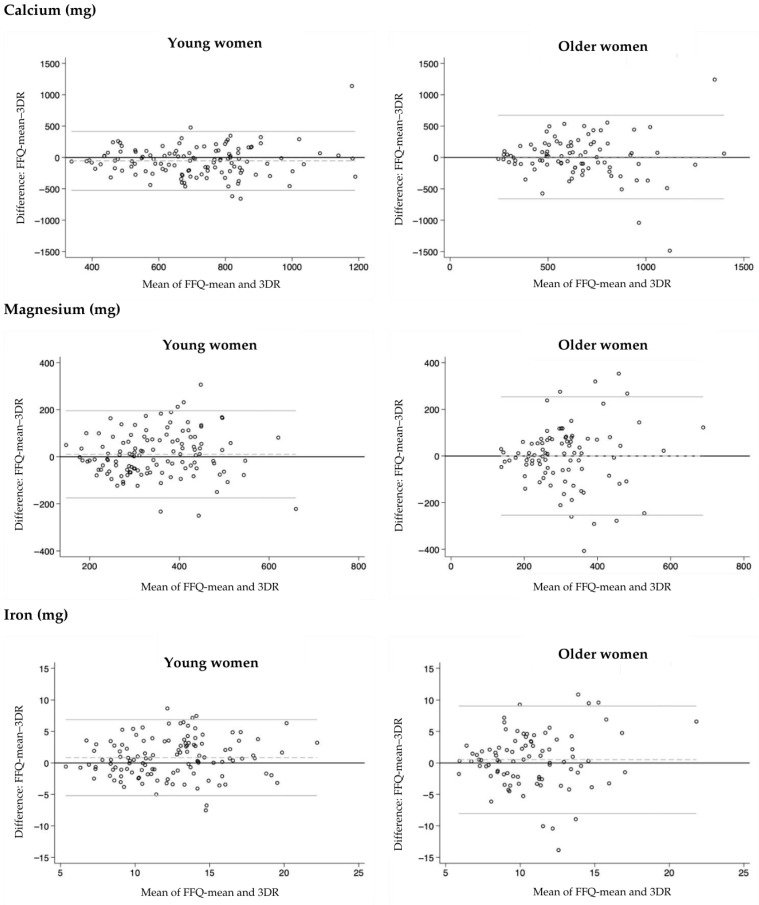
Bland–Altman plots for calcium, magnesium, and iron in young and older women. The plots display the agreement between the FFQ-mean and 3-day diet record (3DR) method for selected micronutrients. Each scatter plot is constructed with the mean of FFQ-mean and 3DR on the x-axis and the difference between FFQ-mean and 3DR on y-axis. The dashed line represents the mean difference in intake between the two methods, while the solid thin lines indicate the 95% limits of agreement.

Bland–Altman analysis was also used to evaluate the agreement between the two methods (Table 4 and Table 5). Regardless of age group, the mean differences were within acceptable ranges for most nutrients, indicating a good overall agreement between the methods. The limits of agreement were relatively narrow for most of the nutrients. Bland–Altman plots did not show clear trends of under- or overestimation by FFQ-mean in either age group, and no potential systematic patterns were identified. Plots for vitamins (vitamin D, folate, vitamin B12) and minerals (calcium, magnesium, iron), which are particularly important for women, are presented in Figure 3 and Figure 4, respectively. For vitamin D and cobalamin, some degree of heteroscedasticity was observed in the plots, indicating that the differences between methods tended to increase at higher intake values. To evaluate the quality of our validation study, Step 1 of the EURECCA tool was used [49]. Our validation received a score of 5 out of 7 points, indicating the good/excellent quality of the validation. Points were awarded for sample size and heterogeneity (1/1 point), statistical assessment (3/3 points), and data collection procedure (1/1 point), while no points were assigned for consideration of seasonality (0/0.5 points) and supplement use (0/1.5 points).

## 4. Discussion

In this study, we assessed the reproducibility and validity of the 138-item FFQ-NutriForHer among 121 young (18–30 years) and 88 older (70–90 years) Polish women. The average reproducibility for macro- and micronutrients, measured using Energy-adjusted PCCs and ICCs, indicated moderate agreement between twice-repeated FFQs. The average validity, assessed using Energy-Adjusted & De-Attenuated Pearson correlation coefficients, demonstrated good concordance between the FFQ-mean and the reference method for both micro- and macronutrients among young women and for macronutrients among older women. To the best of our knowledge, it is the first FFQ designed, developed, and validated specifically to measure dietary intake among women, covering early and older adulthood.

Reproducibility refers to the consistency of questionnaire measurements administered more than once to the same person at different times and constitutes a fundamental element in evaluating new tools [50]. In this study, the mean Energy-adjusted PCCs and ICCs between FFQ1 and FFQ2 were generally slightly higher among young than older women, for both macronutrients (PCC: 0.69 ± 0.12 vs. 0.67 ± 0.06 and ICC: 0.69 ± 0.11 vs. 0.65 ± 0.11, respectively) and micronutrients (PCC: 0.72 ± 0.05 vs. 0.69 ± 0.05 and ICC: 0.71 ± 0.05 vs. 0.69 ± 0.09, respectively). These results are in line with a meta-analysis on the reproducibility of FFQs, which included a total of 123 studies and 20,542 participants [31]. Authors of the meta-analysis reported that ICC values for participants >50 years were lower than for those 18–50 years old for 22 of 42 nutrients. Furthermore, in our study, the range of Energy-adjusted ICC was comparable to the meta-analysis results [31]. Among young women, Energy-adjusted ICC values ranged from 0.57 (fat) to 0.84 (cholesterol), while in the meta-analysis, they ranged from 0.50 (fat) to 0.77 (alcohol) among participants aged 18–50 years. Similarly, among the older, Energy-adjusted ICC values in our study ranged from 0.48 (vitamin A) to 0.84 (magnesium), while in the meta-analysis, values in participants >50 years ranged from 0.50 (dietary fiber) to 0.80 (pyridoxine). The authors of the meta-analysis (based on 123 studies) concluded that correlation coefficients greater than 0.5 for all nutrients may indicate a reliable dietary assessment tool [31]. Since the correlation coefficients for the reliability of FFQ-NutriForHer exceeded these thresholds, it can be considered a reliable tool for measuring dietary intake.

The validity of dietary questionnaires refers to the extent to which the FFQ accurately measures the aspect of diet it was designed to measure [50]. In our study, the means of Energy-adjusted & De-attenuated PCC did not differ substantially between younger and older women for macronutrients (0.51 ± 0.25 vs. 0.46 ± 0.13, respectively). However, a more pronounced difference was observed for micronutrients (0.63 ± 0.13 vs. 0.44 ± 0.14, respectively). This discrepancy may be due to older women having greater difficulty recalling and estimating specific foods rich in micronutrients, whereas younger women may demonstrate better dietary awareness and recall accuracy [51,52,53]. Interestingly, among older women, the Energy-adjusted & De-attenuated PCCs remained relatively similar for both macronutrients and micronutrients. In contrast, among younger women, the observed correlations for macronutrients were lower than those for micronutrients. This may suggest a potential reporting bias in FFQ responses among young women, where they might underreport staple and sweet foods, possibly due to a desire to appear as though they are consuming a healthier or more diverse diet, as observed in previous studies [54,55,56].

Energy adjustment and de-attenuation led to decreased PCC values for most of the nutrients among young women but increased them among older women. A possible explanation could be that young women’s diet may be more variable due to lifestyle factors, characterized by different dietary habits, and reported less accurately compared to those of older women. Older women tend to have more stable diets and less changing diet preferences, so de-attenuation can reveal stronger correlations because nutrient intake is more stable than among younger women [57,58].

Overall, the observed PCC values were similar to those reported in other validation studies among young [59,60,61,62,63] and older people [60,64,65]. However, few studies have reported Energy-adjusted & De-attenuated PCC [59,61,66,67], and none have focused exclusively on women. In our study, among young women, the Energy-adjusted & De-attenuated PCCs ranged from 0.19 (carbohydrates) to 0.86 (vitamin D), while in Canadian participants (22.3 ± 2.5 years; PCC sex, and ethnicity-adjusted) ranged from 0.20 (sodium and polyunsaturated fatty acids) to 0.92 (alcohol) [67], in Korean participants (54.1 ± 7.1 years, 37% women) from 0.36 (iron) to 0.76 (carbohydrates) [61], in Brazilian participants (20–59 years, 62% women) from 0.07 (vitamin E) to 0.64 (cholesterol) [60]. In our study, among older women, the Energy-adjusted & De-attenuated PCCs ranged from 0.24 (thiamine) to 0.77 (vitamin D). However, only one study has specifically reported results for older participants [60]. That study, conducted among the Brazilian participants (60–90 years, 68% women), found Energy-Adjusted and & De-attenuated correlation coefficients ranging from 0.25 (vitamin E) to 0.77 (vitamin C).

Considering the correlation coefficients for nutrients that are particularly important for women (such as vitamin D, folate, cobalamin, calcium, magnesium, and iron), the validation showed that, among young women, most of the nutrients had Energy-Adjusted & De-Attenuated PCCs above 0.7, indicating high validity. However, folate and calcium (PCC = 0.58 and 0.57, respectively) showed moderate validity. In older women, the coefficients for these nutrients were generally slightly lower than in the younger group, but still indicated high validity for vitamin D, moderate validity for calcium and magnesium, and low validity for iron and folate. However, it is important to note that, among older women, iron and folate are generally less critical nutrients compared to young women, primarily due to the absence of menstruation. Additionally, for vitamin D, folate, cobalamin, calcium, magnesium, and iron, correct classification into the same or adjacent quartiles using the FFQ and 3DR method was achieved at least 77% of the time in young women and at least 70% of the time in older women. Furthermore, the Bland–Altman analysis did not show any clear trends of under- or overestimation by the FFQ. An observed degree of heteroscedasticity in the plots for vitamin D and cobalamin is common in dietary assessment research and may reflect true variability in nutrient intake. In particular, lower intake levels tend to show smaller differences between methods, while higher intake levels are typically associated with greater dispersion [68,69]. These findings indicate that the FFQ-NutriForHer can be considered a valid tool for assessing dietary intake in women across different age groups. Given the critical importance of accurate dietary assessment in evaluating health risks, the FFQ-NutriForHer may serve as a reliable instrument in nutritional research, particularly in epidemiological studies focused on assessing nutritional intake and its association with specific women’s health conditions.

The main strength of this study is that the FFQ-NutriForHer addresses a gap identified in the literature by providing one of the few women-specific diet assessment tools developed and validated in a European population, and to our knowledge, it is the first FFQ explicitly designed to measure nutrient intake in women, covering the extreme range of adulthood, from early adulthood (18–30 years) to older adulthood (70–90 years). The FFQ-NutriForHer considers women’s nutritional needs, making it a valuable and reliable tool for research on the associations between diet and women’s health. The study also benefits from a comprehensive statistical analysis, including energy adjustment and de-attenuation validation in both young and older women. Due to reasonable reproducibility and validity in both groups, we hypothesize that the FFQ-NutriForHer can be applied and used to compare dietary intake across different adult age groups, including middle-aged women. However, additional validation in middle-aged women would be valuable to further support the applicability and generalizability of the questionnaire. The results, which showed reasonable reproducibility and validity even among older women, highlight the potential of this tool for large-scale studies. The user-friendly design of the FFQ-NutriForHer may contribute to increased response rates, particularly among older participants who are typically challenging to recruit for nutritional studies. However, it should be noted that for older women with significantly impaired cognitive function, this tool may not be suitable due to challenges in accurately estimating dietary intake over the past year [70].

This study also has some limitations. First, we did not include an analysis of biochemical indicators of consumption, which are often considered a gold standard for assessing validity. However, according to Willett and Lenart [47], these markers seem to be only potentially useful, as reliable biochemical markers reflecting the intake of dietary nutrients are lacking, or existing ones do not provide a quantitative measure of dietary intake, serving rather as qualitative indicators. Second, we did not account for the use of dietary supplements, which can considerably increase nutrient intake. Third, the sample size could have been larger, but according to the meta-analysis by Cui et al. [31], the median sample size in 111 validation studies, including both women and men, was 102 among adults (18–50 years) and 158 among older adults (above 50 years). It is worth noting that in our study, the older women were much older than 50 years, with an average age of 74.7 ± 5.2 years. The authors of the meta-analysis [31] concluded that “a sample size with sufficient statistical power, but no larger, is recommended for validation studies” as larger samples do not necessarily enhance reproducibility. In our study, the sample sizes provided high statistical power (>0.97) to detect correlations considered reliable by Cui et al. [31] (r > 0.5) in FFQ validation studies. However, the ability to detect weaker correlations (e.g., r ≈ 0.3) was more limited, particularly in the older women’s group (statistical power 0.81), although it remained above the generally accepted threshold of 0.8 [71]. Therefore, although the sample size met recommended standards and was adequate for detecting moderate-to-strong correlations, the limited power for weaker relationships should be acknowledged as a potential limitation.

Moreover, the older women recruited for this study were mostly from large cities and senior clubs, where members tend to have better cognitive function. Furthermore, we did not include middle-aged women in this study. This, along with the use of convenience sampling, may limit the generalizability of the findings. However, according to Step 1, Point 1, “Sample and sample size” of the EURRECA tool for evaluating the quality of validation studies [49], 0.5 points were assigned for the sample size because our study included more than 100 individuals, comprising a total of 209 women (121 younger and 88 older). Another limitation is that 3DRs were collected only once. Repeated diet records over multiple time points could have provided more accurate dietary intake data and improved the validation; however, such an approach could be too burdensome for older women. Finally, the list of food items in the FFQ-NutriForHer is based on European food products, which may limit its applicability in culturally distinct regions with different food profiles, such as Asia or Africa.

## 5. Conclusions

Given the high reproducibility and satisfactory validity in both age groups, the FFQ-NutriForHer is a reliable tool for assessing dietary intake and examining its links to women’s health across different ages. In future revisions of the FFQ-NutriForHer, incorporating a dedicated item addressing the use of dietary supplements (e.g., vitamins and minerals) would enhance the comprehensiveness of the assessment by capturing additional sources of nutrient intake. Moreover, conducting a validation study among middle-aged women and developing an online version of the questionnaire would further strengthen its applicability, generalizability, and accessibility.

## Figures and Tables

**Table 1:** Characteristics of young (18–30 years old) and older (70–90 years old) women.

Characteristics	Young Women n = 121		Older Women n = 88		*p*-Value Chi^2^ Test
	n	%	n	%	
Age, years	22.6 ± 1.6		74.7 ± 5.1		
Body mass index, kg/m^2^					
<18.5	9	7.4	3	3.4	
18.5–24.9	105	86.8	39	44.3	<0.001
≥25.0	7	5.8	46	52.3	
Place of living					
Village	33	27.3	15	17.0	
Town (<100,000 residents)	19	15.7	14	15.9	0.21
City (>100,000 residents)	69	57.0	59	67.1	
Economic status (self-assessment)					
Poor	2	1.7	6	6.8	
Average	37	30.6	39	44.3	0.01
Good/very good	82	67.7	43	48.9	
Smoking status					
Never	94	77.7	43	48.9	
Former smokers	20	16.5	35	39.8	<0.001
Current smokers	7	5.8	10	11.3	
Health status (self-assessment)					
Poor	2	1.7	4	4.6	
Average	24	19.8	39	44.3	<0.001
Good	47	38.8	39	44.3	
Very good	48	39.7	6	6.8	
Physical activity (self-assessment)					
Very low/low	35	28.9	55	62.5	
Moderate	67	55.4	27	30.7	<0.001
High/very high	19	15.7	6	6.8	

**Table 2:** Reproducibility of the food frequency questionnaire (FFQ-NutriForHer) for energy and macronutrient intake among young and older women.

Parameter	FFQ1		FFQ2		PCC		ICC	
	Median	P25–P75	Median	P25–P75	Crude	Energy- Adjusted *	Crude	Energy- Adjusted *
YOUNG WOMEN (n = 121)								
Energy, kcal	1650	1364–1927	1567	1237–1809	0.71	-	0.68	-
Protein, g	66.9	52.8–81.9	64.2	49.4–76.8	0.81	0.77	0.78	0.75
Carbohydrate, g	202	173–241	194	153–228	0.64	0.55	0.61	0.59
Dietary fiber, g	23.4	17.5–32.3	21.0	14.9–28.3	0.73	0.71	0.70	0.72
Fat, g	60.0	46.5–73.3	54.0	42.6–69.3	0.66	0.59	0.65	0.57
Cholesterol, mg	230	149–320	229	146–325	0.84	0.84	0.82	0.84
MEAN ± SD ^#^	-	-	-	-	0.74 ± 0.09	0.69 ± 0.12	0.71 ± 0.09	0.69 ± 0.11
OLDER WOMEN (n = 88)								
Energy, kcal	1627	1223–2059	1497	1278–1901	0.67	-	0.66	-
Protein, g	67.6	48.8–86.3	64.1	47.5–76.1	0.69	0.70	0.68	0.74
Carbohydrate, g	201	157–258	198	154–238	0.70	0.69	0.69	0.64
Dietary fiber, g	20.6	13.9–27.6	19.6	12.6–26.0	0.75	0.74	0.75	0.78
Fat, g	63.6	42.1–82.7	61.8	44.4–79.1	0.60	0.59	0.60	0.51
Cholesterol, mg	240	176–296	229	182–283	0.61	0.62	0.61	0.59
MEAN ± SD ^#^	-	-	-	-	0.67 ± 0.06	0.67 ± 0.06	0.67 ± 0.06	0.65 ± 0.11

Abbreviations: FFQ1, food frequency questionnaire 1; FFQ2, food frequency questionnaire 2; P25–P75, the values of 25th and 75th percentiles; PCC, Pearson correlation coefficient; ICC, intraclass correlation coefficient. * Energy-adjusted to the mean energy intake assessed by the 3-day record method (1642 kcal/day in young women and 1614 kcal/day in older women) using the residual method (Willett and Stampfer, 1986 [42]). ^#^ The means are based on protein, carbohydrates, dietary fiber, fat, and cholesterol.

**Table 3:** Reproducibility of the food frequency questionnaire (FFQ-NutriForHer) for micronutrient intake among young and older women.

Parameter	FFQ1		FFQ2		PCC		ICC	
	Median	P25–P75	Median	P25–P75	Crude	Energy- Adjusted *	Crude	Energy- Adjusted *
YOUNG WOMEN (n = 121)								
Vitamin A, µg RE	1102	844–1636	1063	722–1496	0.65	0.66	0.64	0.69
Vitamin E, mg TE	12.7	9.5–17.8	12.2	7.7–16.0	0.71	0.68	0.68	0.68
Vitamin D, µg	2.86	1.83–4.32	3.02	1.75–4.48	0.77	0.76	0.76	0.72
Thiamine, mg	1.10	0.90–1.36	1.07	0.73–1.29	0.73	0.70	0.70	0.66
Riboflavin, mg	1.52	1.20–1.90	1.42	1.10–1.78	0.74	0.71	0.71	0.72
Folate, µg DFE	339	258–438	307	237–411	0.70	0.69	0.67	0.75
Pyridoxine, mg	2.00	1.54–2.51	1.81	1.38–2.31	0.75	0.71	0.72	0.70
Cobalamin, µg	3.66	2.72–5.29	3.64	2.47–5.09	0.79	0.78	0.78	0.78
Vitamin C, mg	132	99–190	132	84–180	0.61	0.62	0.58	0.63
Calcium, mg	655	531–852	642	483–796	0.69	0.68	0.67	0.67
Phosphorus, mg	1222	983–1532	1152	887–1433	0.80	0.77	0.78	0.71
Magnesium, mg	363	272–461	328	242–419	0.80	0.78	0.78	0.81
Iron, mg	13.0	10.1–15.6	12.0	8.9–15.2	0.75	0.72	0.72	0.70
Zinc, mg	9.5	7.7–11.6	9.0	6.8–11.1	0.78	0.75	0.76	0.72
MEAN ± SD	-	-	-	-	0.73 ± 0.06	0.72 ± 0.05	0.71 ± 0.06	0.71 ± 0.05
OLDER WOMEN (n = 88)								
Vitamin A, µg RE	1179	670–1740	1054	782–1567	0.59	0.59	0.58	0.48
Vitamin E, mg TE	11.1	7.0–17.5	11.0	8.4–16.2	0.71	0.71	0.70	0.65
Vitamin D, µg	3.3	1.9–5.9	3.2	2.16–5.46	0.59	0.59	0.58	0.58
Thiamine, mg	1.0	0.8–1.4	0.94	0.68–1.13	0.70	0.71	0.69	0.75
Riboflavin, mg	1.5	1.1–1.9	1.46	1.08–1.68	0.69	0.69	0.67	0.72
Folate, µg DFE	281	231–377	280	211–356	0.72	0.72	0.71	0.68
Pyridoxine, mg	1.6	1.2–2.0	1.59	1.11–2.07	0.69	0.70	0.69	0.70
Cobalamin, µg	4.3	2.5–6.5	3.90	2.55–5.78	0.69	0.69	0.68	0.69
Vitamin C, mg	106	67–172	111	72–169	0.67	0.67	0.67	0.66
Calcium, mg	638	448–800	598	405–787	0.75	0.75	0.74	0.78
Phosphorus, mg	1179	860–1475	1089	770–1299	0.73	0.73	0.71	0.77
Magnesium, mg	299	219–388	285	206–359	0.79	0.78	0.78	0.84
Iron, mg	10.6	8.2–14.1	9.8	7.7–13.0	0.69	0.69	0.68	0.72
Zinc, mg	8.9	6.6–11.2	8.1	6.1–10.1	0.70	0.70	0.68	0.70
MEAN ± SD	-	-	-	-	0.69 ± 0.05	0.69 ± 0.05	0.68 ± 0.05	0.69 ± 0.09

Abbreviations: FFQ1, food frequency questionnaire 1; FFQ2, food frequency questionnaire 2; P25–P75, the values of 25th and 75th percentiles; PCC, Pearson correlation coefficient; ICC, intraclass correlation coefficient; DFE, dietary folate equivalents; RE, retinol equivalents; TE, α–tocopherol equivalents. * Energy-adjusted to the mean energy intake assessed by the 3-day record method (1642 kcal/day in young women and 1614 kcal/day in older women) using the residual method (Willett and Stampfer, 1986 [42]).

**Table 4:** Relative validity of the food frequency questionnaire (FFQ-NutriForHer) for energy and macronutrient intake among young and older women.

Parameter	FFQ-Mean		3-Day Food Record		PCC				Identical or Adjacent Quartile (%) ^	Opposite Quartile (%) ^	Bland–Altman ^	
	Median	P25–P75	Median	P25–P75	Crude	Energy- Adjusted *	De- Attenuated	Energy- Adjusted * & De- Attenuated			Mean Difference	LOA
YOUNG WOMEN (n = 121)												
Energy, kcal	1604	1356–1855	1635	1445–1831	0.55	-	0.73	-	78.5	2.5	−41.5	−679 to 596
Protein, g	63.8	51.9–78.2	69.4	60.2–81.3	0.59	0.47	0.76	0.59	79.3	2.5	−3.4	−39 to 32
Carbohydrates, g	200	167–231	209	181–232	0.46	0.16	0.57	0.19	76.9	7.4	−7.7	−105 to 91
Dietary fiber, g	22.6	16.4–29.5	20.0	14.8–24.7	0.58	0.54	0.67	0.62	84.3	3.3	3.0	−11 to 17
Fat, g	57.7	45.0–71.3	58.3	50.8–69.3	0.48	0.23	0.66	0.31	82.7	4.1	−0.9	−36 to 34
Cholesterol, mg	228	155–316	271	189–361	0.56	0.55	0.79	0.82	83.5	0	−37.2	−258 to 184
MEAN ± SD ^#^	-	-	-	-	0.53 ± 0.06	0.39 ± 0.18	0.69 ± 0.09	0.51 ± 0.25	81.3 ± 3.1	3.46 ± 2.7	-	-
OLDER WOMEN (n = 88)												
Energy, kcal	1531	1292–1965	1568	1272–1898	0.21	-	0.24	-	73.9	6.8	39.6	−1126 to 1205
Protein, g	63.2	52.3–77.9	68.3	54.3–80.1	0.23	0.29	0.28	0.34	75.0	5.7	−0.9	−49 to 48
Carbohydrates, g	200.3	157–247	209.6	163–241	0.31	0.32	0.35	0.40	75.0	8.0	−0.6	−156 to 155
Dietary fiber, g	19.5	14.6–26.8	18.2	13.8–23.9	0.43	0.42	0.48	0.50	80.7	2.3	2.4	−17 to 22
Fat, g	62.4	44.4–82.0	59.7	42.0–74.2	0.37	0.32	0.43	0.40	80.7	5.7	5.2	−49 to 59
Cholesterol, mg	238.2	184–282	261.7	181–327	0.24	0.26	0.47	0.67	70.5	5.7	−18.3	−279 to 242
MEAN ± SD ^#^	-	-	-	-	0.32 ± 0.09	0.32 ± 0.06	0.40 ± 0.09	0.46 ± 0.13	76.4 ± 4.4	5.48 ± 2.0	-	-

Abbreviations: P25–P75, the values of 25th and 75th percentiles; PCC, Pearson correlation coefficient; LOA, limits of agreement. * Energy-adjusted to the mean energy intake assessed by the 3-day record method (1642 kcal/day in young women and 1614 kcal/day in older women) using the residual method (Willett and Stampfer, 1986 [42]). ^#^ The means are based on protein, carbohydrates, dietary fiber, fat, and cholesterol. ^ Calculated using crude variables.

**Table 5:** Relative validity of the food frequency questionnaire (FFQ-NutriForHer) for micronutrient intake among young and older women.

Parameter	FFQ-Mean		3-Day Food Record		PCC				Identical or Adjacent Quartile (%) ^	Opposite Quartile (%) ^	Bland–Altman ^	
	Median	P25–P75	Median	P25–P75	Crude	Energy- Adjusted *	De- Attenuated	Energy- Adjusted * & De-Attenuated			Mean Difference	LOA
YOUNG WOMEN (n = 121)												
Vitamin A, µg RE	1110	827–1582	1017	679–1446	0.34	0.34	0.44	0.44	76.9	5.8	195	−1256 to 1646
Vitamin E, mg TE	12.3	9.3–16.4	11.4	8.8–14.4	0.58	0.49	0.74	0.65	83.5	2.5	1.7	−6.6 to 10.1
Vitamin D, µg	2.99	1. 91–4.31	2.28	1.42–3.37	0.50	0.47	0.85	0.86	79.9	2.5	0.44	−3.93 to 4.81
Thiamine, mg	1.08	0.83–1.29	0.96	0.86–1.19	0.50	0.37	0.65	0.51	81.0	5.8	0.06	−0.56 to 0.68
Riboflavin, mg	1.50	1.13–1.83	1.64	1.35–1.85	0.62	0.56	0.85	0.71	84.3	3.3	−0.09	−0.88 to 0.69
Folate, µg DFE	322	262–423	311	244–405	0.53	0.51	0.61	0.58	80.8	0.8	19	−196 to 234
Pyridoxine, mg	1.95	1.50–2.40	1.84	1.38–2.24	0.47	0.35	0.57	0.43	77.7	7.4	0.18	−1.1 to 1.5
Cobalamin, µg	3.90	2.66–5.00	3.08	2.40–4.05	0.52	0.50	0.87	0.84	76.9	2.5	0.78	−3.3 to 4.9
Vitamin C, mg	135	94–170	108	72–162	0.41	0.41	0.50	0.50	76.9	4.1	22	−136 to 180
Calcium, mg	664	532–822	722	594–862	0.45	0.43	0.71	0.57	78.5	5.0	−54	−523 to 414
Phosphorus, mg	1220	972–1498	1271	1030–1429	0.63	0.55	0.79	0.65	85.1	2.5	−29	−620 to 562
Magnesium, mg	337	258–449	324	274–410	0.64	0.60	0.74	0.69	84.3	1.7	10.3	−175 to 196
Iron, mg	12.3	9.9–15.4	11.6	9.5–14.1	0.65	0.58	0.80	0.71	84.3	1.7	0.9	−5.2 to 6.9
Zinc, mg	9.2	7.4–11.2	9.2	7.7–10.8	0.61	0.52	0.75	0.62	86.8	4.1	0.04	−4.5 to 4.6
MEAN ± SD	-	-	-	-	0.53 ± 0.09	0.48 ± 0.09	0.71 ± 0.13	0.63 ± 0.13	81.2 ± 3.5	3.55 ± 1.9	-	-
OLDER WOMEN (n = 88)												
Vitamin A, µg RE	1210	798–1583	963	618–1300	0.22	0.23	0.31	0.35	73.8	11.4	298	−1560 to 2156
Vitamin E, mg TE	11.1	8.0–16.9	8.7	5.8–11.5	0.40	0.37	0.47	0.49	75.0	4.6	3.1	−8.9 to 15.1
Vitamin D, µg	3.43	2.11–5.29	2.30	1.53–4.55	0.41	0.41	0.73	0.77	73.9	4.4	0.51	−5.64 to 6.66
Thiamine, mg	0.99	0.76–1.17	0.89	0.74–1.16	0.15	0.17	0.18	0.24	69.3	8.0	0.05	−0.88 to 0.98
Riboflavin, mg	1.48	1.16–1.75	1.50	1.19–1.77	0.33	0.35	0.43	0.51	71.6	5.7	0.01	−1.05 to 1.07
Folate, µg DFE	287	222–363	264	186–329	0.20	0.22	0.22	0.27	71.6	9.1	31	−236 to 298
Pyridoxine, mg	1.55	1.24–1.95	1.59	1.18–1.81	0.25	0.27	0.31	0.35	65.9	10.2	0.11	−1.3 to 1.5
Cobalamin, µg	4.23	2.63–6.44	2.85	1.96–4.36	0.39	0.40	0.50	0.50	75.0	5.7	1.33	−6.4 to 9.1
Vitamin C, mg	115	74–161	74	49–122	0.46	0.44	0.53	0.54	79.6	6.8	33	−151 to 218
Calcium, mg	622	451–782	579	430–785	0.44	0.44	0.51	0.53	78.4	4.6	7	−659 to 674
Phosphorus, mg	1151	867–1361	1137	853–1315	0.34	0.37	0.39	0.41	72.7	6.8	15	−850 to 880
Magnesium, mg	283	223–366	286	231–365	0.45	0.44	0.50	0.49	76.1	5.7	−0.3	−254 to 253
Iron, mg	10.4	7.8–12.9	9.9	8.0–12.5	0.23	0.22	0.28	0.32	70.4	5.7	0.5	−8.0 to 9.1
Zinc, mg	8.6	6.7–10.2	8.6	7.0–9.9	0.26	0.31	0.31	0.39	73.9	8.0	−0.07	−6.4 to 6.2
MEAN ± SD	-	-	-	-	0.32 ± 0.10	0.33 ± 0.09	0.41 ± 0.15	0.44 ± 0.14	73.4 ± 3.6	6.91 ± 2.2	-	-

Abbreviations: P25–P75, the values of 25th and 75th percentiles; PCC, Pearson correlation coefficient; LOA, limits of agreement; DFE, dietary folate equivalents; RE, retinol equivalents; TE, α–tocopherol equivalents. * Energy-adjusted to the mean energy intake assessed by the 3-day record method (1642 kcal/day in young women and 1614 kcal/day in older women) using the residual method (Willett and Stampfer, 1986 [42]). ^ Calculated using crude variables.

## Data Availability

The datasets generated for this study are available from the corresponding author upon reasonable request.

## Supplementary Materials

The following supporting information can be downloaded at https://www.mdpi.com/article/10.3390/nu17243811/s1, Supplementary S1: FFQ-NutriForHer—English version; Supplementary S2: FFQ—NutriForHer—Polish version.

## Abbreviations

The following abbreviations are used in this manuscript:

3DR	3-day diet record
FFQ	Food Frequency Questionnaire
FFQ1	Food Frequency Questionnaire 1
FFQ2	Food Frequency Questionnaire 2
FFQ-mean	mean value of two food frequency questionnaires
ICC	intra-class correlation coefficient
PPC	Pearson Correlation Coefficient
Q1, Q2, Q3, Q4	quartile 1, quartile 2, quartile 3, quartile 4
