# Supplementary material for: FFQ-NutriForHer: Reproducibility and Validity of a Semi-Quantitative Food Frequency Questionnaire for Young and Older Women
Source: Nutrients. 2025 Dec 5;17(24):3811. doi: 10.3390/nu17243811 (PMC12735852; doi:10.3390/nu17243811)
Supplement: Supplementary file 1 [file nutrients-17-03811-s001.zip › Supplementary S1.pdf]

<u>Supplementary S1</u>

FFQ-NutriForHer:

Food Frequency Questionnaire on selected food products consumption <u>in the last year</u>

ID OF PARTICIPANT: __________

DATE: _____________________

Instruction:

Please indicate how often you consumed the indicated product **<u>in the last year</u>** by **checking the appropriate box ☒** and please provide **a portion of the product consumed on average <u>at one time.</u>**

Example:

**Products**	**Times per month**		**per week**			**per day**			**Amount** (g or ml) **or portion size** (number of pieces, slices, teaspoons, etc.)
	**<1**	**1-3**	**1-2**	**3-4**	**5-6**	**1**	**2**	**3+**	
**Cereal products**									
White bread and rolls	☐	☒	☐	☐	☐	☐	☐	☐	*1 kaiser roll*
Whole grain bread	☐	☐	☐	☐	☐	☐	☒	☐	*1 thick slice*
Crispbread	☒	☐	☐	☐	☐	☐	☐	☐	____________
White pasta	☐	☒	☐	☐	☐	☐	☐	☐	*100 g, cooked*
Wholemeal pasta	☐	☐	☒	☐	☐	☐	☐	☐	*1 cup, dry*
White rice	☐	☐	☐	☒	☐	☐	☐	☐	*4 tablespoons, cooked*

Food frequency questionnaire (FFQ-NutriForHer)

**Products**	**Times per month**		**per week**			**per day**			**Amount** (g or ml) **or portion size** (number of pieces, slices, teaspoons, etc.)
	**<1**	**1-3**	**1-2**	**3-4**	**5-6**	**1**	**2**	**3+**	
**Cereal products**									
White bread and rolls	☐	☐	☐	☐	☐	☐	☐	☐	____________
Whole grain bread	☐	☐	☐	☐	☐	☐	☐	☐	____________
Crispbread/rice waffles	☐	☐	☐	☐	☐	☐	☐	☐	____________
White pasta	☐	☐	☐	☐	☐	☐	☐	☐	____________
Wholemeal pasta	☐	☐	☐	☐	☐	☐	☐	☐	____________
White rice	☐	☐	☐	☐	☐	☐	☐	☐	____________
Brown rice	☐	☐	☐	☐	☐	☐	☐	☐	____________
Fine groats (semolina, Mazurian, couscous)	☐	☐	☐	☐	☐	☐	☐	☐	____________
Coarse groats (buckwheat, hulled barley, millet)	☐	☐	☐	☐	☐	☐	☐	☐	____________
Sweetened breakfast cereal	☐	☐	☐	☐	☐	☐	☐	☐	____________
Natural cereal flakes/muesli	☐	☐	☐	☐	☐	☐	☐	☐	____________
Wheat/oat bran	☐	☐	☐	☐	☐	☐	☐	☐	____________
**Milk products**									
Skimmed milk (0-0,5% fat)	☐	☐	☐	☐	☐	☐	☐	☐	____________
Semi-skimmed milk (1,5-2% fat)	☐	☐	☐	☐	☐	☐	☐	☐	____________
Whole milk (3,2-3,5% fat)	☐	☐	☐	☐	☐	☐	☐	☐	____________
Kefir/yoghurt (0% fat)	☐	☐	☐	☐	☐	☐	☐	☐	____________
Kefir/yoghurt (1,5-2% fat)	☐	☐	☐	☐	☐	☐	☐	☐	____________
Quark cheese (full-fat)	☐	☐	☐	☐	☐	☐	☐	☐	____________
Quark cheese (low-fat/semi-fat)	☐	☐	☐	☐	☐	☐	☐	☐	____________
Cream cheese	☐	☐	☐	☐	☐	☐	☐	☐	____________
Hard cheese	☐	☐	☐	☐	☐	☐	☐	☐	____________
Blue cheese	☐	☐	☐	☐	☐	☐	☐	☐	____________
Salad cheese (feta, mozzarella)	☐	☐	☐	☐	☐	☐	☐	☐	____________
Plant-based milks	☐	☐	☐	☐	☐	☐	☐	☐	____________
**Meat and poultry**									
Pork (including minced meat)	☐	☐	☐	☐	☐	☐	☐	☐	____________
Beef/veal (including minced meat)	☐	☐	☐	☐	☐	☐	☐	☐	____________
Chicken/turkey (including minced meat)	☐	☐	☐	☐	☐	☐	☐	☐	____________
Liver/other offal	☐	☐	☐	☐	☐	☐	☐	☐	____________
Poultry ham/cold meat	☐	☐	☐	☐	☐	☐	☐	☐	____________
Pork ham/cold meat	☐	☐	☐	☐	☐	☐	☐	☐	____________

<u>Food frequency questionnaire (FFQ-NutriForHer)</u>

**Products**	**Times per month**		**per week**			**per day**			**Amount** (g or ml) **or portion size** (number of pieces, slices, teaspoons, etc.)
	**<1**	**1-3**	**1-2**	**3-4**	**5-6**	**1**	**2**	**3+**	
Sausages	☐	☐	☐	☐	☐	☐	☐	☐	____________
Vienna sausages	☐	☐	☐	☐	☐	☐	☐	☐	____________
Salami/bacon/luncheon meat	☐	☐	☐	☐	☐	☐	☐	☐	____________
Pâté	☐	☐	☐	☐	☐	☐	☐	☐	____________
**Eggs, fish and seafood**									
Eggs	☐	☐	☐	☐	☐	☐	☐	☐	____________
Herring	☐	☐	☐	☐	☐	☐	☐	☐	____________
Mackerel	☐	☐	☐	☐	☐	☐	☐	☐	____________
Salmon	☐	☐	☐	☐	☐	☐	☐	☐	____________
Tuna	☐	☐	☐	☐	☐	☐	☐	☐	____________
Sardines	☐	☐	☐	☐	☐	☐	☐	☐	____________
Cod	☐	☐	☐	☐	☐	☐	☐	☐	____________
Pollock	☐	☐	☐	☐	☐	☐	☐	☐	____________
Other fish: _________________	☐	☐	☐	☐	☐	☐	☐	☐	____________
Shrimp	☐	☐	☐	☐	☐	☐	☐	☐	____________
Other seafoods: __________	☐	☐	☐	☐	☐	☐	☐	☐	____________
**Vegetables**									
Lettuce	☐	☐	☐	☐	☐	☐	☐	☐	____________
Spinach	☐	☐	☐	☐	☐	☐	☐	☐	____________
Arugula/lamb's lettuce	☐	☐	☐	☐	☐	☐	☐	☐	____________
Kale	☐	☐	☐	☐	☐	☐	☐	☐	____________
Potatoes	☐	☐	☐	☐	☐	☐	☐	☐	____________
Carrot	☐	☐	☐	☐	☐	☐	☐	☐	____________
Celery/parsley	☐	☐	☐	☐	☐	☐	☐	☐	____________
Onion/leek	☐	☐	☐	☐	☐	☐	☐	☐	____________
Beetroot	☐	☐	☐	☐	☐	☐	☐	☐	____________
Cauliflower	☐	☐	☐	☐	☐	☐	☐	☐	____________
Broccoli	☐	☐	☐	☐	☐	☐	☐	☐	____________
Cabbage (white, red, brussels sprouts)	☐	☐	☐	☐	☐	☐	☐	☐	____________
Pickled vegetables (cabbage/cucumbers)	☐	☐	☐	☐	☐	☐	☐	☐	____________
Pepper	☐	☐	☐	☐	☐	☐	☐	☐	____________
Tomato	☐	☐	☐	☐	☐	☐	☐	☐	____________
Green beans	☐	☐	☐	☐	☐	☐	☐	☐	____________
Green peas	☐	☐	☐	☐	☐	☐	☐	☐	____________

Food frequency questionnaire (FFQ-NutriForHer)

Products	Times per month <1	1-3	per week 1-2	3-4	5-6	per day 1	2	3+	**Amount** (g or ml) **or portion size** (number of pieces, slices, teaspoons, etc.)
Pumpkin	☐	☐	☐	☐	☐	☐	☐	☐	____________
Corn	☐	☐	☐	☐	☐	☐	☐	☐	____________
Olives	☐	☐	☐	☐	☐	☐	☐	☐	____________
Frozen vegetable mix	☐	☐	☐	☐	☐	☐	☐	☐	____________
Other vegetables_______________	☐	☐	☐	☐	☐	☐	☐	☐	____________
**Fruit**									
Banana	☐	☐	☐	☐	☐	☐	☐	☐	____________
Apple/pear	☐	☐	☐	☐	☐	☐	☐	☐	____________
Orange/grapefruit/mandarin	☐	☐	☐	☐	☐	☐	☐	☐	____________
Kiwi	☐	☐	☐	☐	☐	☐	☐	☐	____________
Peach/apricot	☐	☐	☐	☐	☐	☐	☐	☐	____________
Grapes	☐	☐	☐	☐	☐	☐	☐	☐	____________
Cherries	☐	☐	☐	☐	☐	☐	☐	☐	____________
Plums	☐	☐	☐	☐	☐	☐	☐	☐	____________
Watermelon/melon	☐	☐	☐	☐	☐	☐	☐	☐	____________
Mango/pineapple	☐	☐	☐	☐	☐	☐	☐	☐	____________
Strawberries/raspberries	☐	☐	☐	☐	☐	☐	☐	☐	____________
Blueberries/bilberry/cranberries	☐	☐	☐	☐	☐	☐	☐	☐	____________
Avocado	☐	☐	☐	☐	☐	☐	☐	☐	____________
Dried fruits	☐	☐	☐	☐	☐	☐	☐	☐	____________
**Legumes (dry seeds)**									
Peas	☐	☐	☐	☐	☐	☐	☐	☐	____________
Lentils	☐	☐	☐	☐	☐	☐	☐	☐	____________
Beans	☐	☐	☐	☐	☐	☐	☐	☐	____________
Soybeans/soy products	☐	☐	☐	☐	☐	☐	☐	☐	____________
Chickpeas	☐	☐	☐	☐	☐	☐	☐	☐	____________
**Nuts and seeds**									
Almonds	☐	☐	☐	☐	☐	☐	☐	☐	____________
Hazelnuts	☐	☐	☐	☐	☐	☐	☐	☐	____________
Walnuts	☐	☐	☐	☐	☐	☐	☐	☐	____________
Unsalted peanuts	☐	☐	☐	☐	☐	☐	☐	☐	____________
Pistachios	☐	☐	☐	☐	☐	☐	☐	☐	____________
Salted nuts	☐	☐	☐	☐	☐	☐	☐	☐	____________
Other nuts: _________________	☐	☐	☐	☐	☐	☐	☐	☐	____________

Food frequency questionnaire (FFQ-NutriForHer)

Products	Times per month <1	1-3	per week 1-2	3-4	5-6	per day 1	2	3+	**Amount** (g or ml) **or portion size** (number of pieces, slices, teaspoons, etc.)
Nut and raisin mix	☐	☐	☐	☐	☐	☐	☐	☐	____________
Peanut butter	☐	☐	☐	☐	☐	☐	☐	☐	____________
Sunflower seeds	☐	☐	☐	☐	☐	☐	☐	☐	____________
Pumpkin seeds	☐	☐	☐	☐	☐	☐	☐	☐	____________
Flax seeds	☐	☐	☐	☐	☐	☐	☐	☐	____________
**Sugar and confectionery**									
Sugar	☐	☐	☐	☐	☐	☐	☐	☐	____________
Honey	☐	☐	☐	☐	☐	☐	☐	☐	____________
Jam	☐	☐	☐	☐	☐	☐	☐	☐	____________
Sweetener: __________________	☐	☐	☐	☐	☐	☐	☐	☐	____________
Confectionery bread	☐	☐	☐	☐	☐	☐	☐	☐	____________
Puff pastries	☐	☐	☐	☐	☐	☐	☐	☐	____________
Cookies/biscuits	☐	☐	☐	☐	☐	☐	☐	☐	____________
Milk chocolate	☐	☐	☐	☐	☐	☐	☐	☐	____________
Dark chocolate	☐	☐	☐	☐	☐	☐	☐	☐	____________
Chocolate bars/wafers/candies	☐	☐	☐	☐	☐	☐	☐	☐	____________
Candy/fudge	☐	☐	☐	☐	☐	☐	☐	☐	____________
Ice cream/sorbets	☐	☐	☐	☐	☐	☐	☐	☐	____________
Nutella/chocolate spread	☐	☐	☐	☐	☐	☐	☐	☐	____________
**Non-alcoholic beverages**									
Mineral/spring water	☐	☐	☐	☐	☐	☐	☐	☐	____________
Tea	☐	☐	☐	☐	☐	☐	☐	☐	____________
Coffee	☐	☐	☐	☐	☐	☐	☐	☐	____________
Fruit juices (orange/apple)	☐	☐	☐	☐	☐	☐	☐	☐	____________
Vegetable juices (carrot/tomato)	☐	☐	☐	☐	☐	☐	☐	☐	____________
Other juices___________________	☐	☐	☐	☐	☐	☐	☐	☐	____________
Fruit drinks/compote	☐	☐	☐	☐	☐	☐	☐	☐	____________
Soft drinks (type cola)	☐	☐	☐	☐	☐	☐	☐	☐	____________
Isotonic drinks	☐	☐	☐	☐	☐	☐	☐	☐	____________
Energy drinks	☐	☐	☐	☐	☐	☐	☐	☐	____________
**Alcoholic beverages**									
Beer	☐	☐	☐	☐	☐	☐	☐	☐	____________
White wine/champagne	☐	☐	☐	☐	☐	☐	☐	☐	____________
Red wine	☐	☐	☐	☐	☐	☐	☐	☐	____________

Food frequency questionnaire (FFQ-NutriForHer)

Products	Times per month		per week			per day			**Amount** (g or ml) **or portion size** (number of pieces, slices, teaspoons, etc.)
	**<1**	**1-3**	**1-2**	**3-4**	**5-6**	**1**	**2**	**3+**	
Alcohol-based drinks	☐	☐	☐	☐	☐	☐	☐	☐	____________
Vodka/strong alcohol	☐	☐	☐	☐	☐	☐	☐	☐	____________
Other alcohol: _______________	☐	☐	☐	☐	☐	☐	☐	☐	____________
**Fats and spreads**									
Olive oil	☐	☐	☐	☐	☐	☐	☐	☐	____________
Sunflower oil	☐	☐	☐	☐	☐	☐	☐	☐	____________
Canola oil	☐	☐	☐	☐	☐	☐	☐	☐	____________
Linseed oil	☐	☐	☐	☐	☐	☐	☐	☐	____________
Coconut oil	☐	☐	☐	☐	☐	☐	☐	☐	____________
Butter (82% fat)	☐	☐	☐	☐	☐	☐	☐	☐	____________
Cream (9-18% fat)	☐	☐	☐	☐	☐	☐	☐	☐	____________
Cream (30-36% fat)	☐	☐	☐	☐	☐	☐	☐	☐	____________
Margarine: ____________________	☐	☐	☐	☐	☐	☐	☐	☐	____________
Butter-mixed spread:___________	☐	☐	☐	☐	☐	☐	☐	☐	____________
Lard	☐	☐	☐	☐	☐	☐	☐	☐	____________
Other fats: ____________________	☐	☐	☐	☐	☐	☐	☐	☐	____________
**Other products**									
Crisps/French fries	☐	☐	☐	☐	☐	☐	☐	☐	____________
Popcorn	☐	☐	☐	☐	☐	☐	☐	☐	____________
Pizza/hamburger/kebab	☐	☐	☐	☐	☐	☐	☐	☐	____________
Pancakes/dumplings	☐	☐	☐	☐	☐	☐	☐	☐	____________
Mayonnaise	☐	☐	☐	☐	☐	☐	☐	☐	____________
Ketchup	☐	☐	☐	☐	☐	☐	☐	☐	____________
