# Supplementary material for: FFQ-NutriForHer: Reproducibility and Validity of a Semi-Quantitative Food Frequency Questionnaire for Young and Older Women
Source: Nutrients. 2025 Dec 5;17(24):3811. doi: 10.3390/nu17243811 (PMC12735852; doi:10.3390/nu17243811)
Supplement: Supplementary file 1 [file nutrients-17-03811-s001.zip › Supplementary S2.pdf]

Supplementary S2

FFQ-NutriForHer:
Kwestionariusz częstotliwości spożycia wybranych produktów w ostatnim roku

NUMER RESPONDENTA: _______________

DATA: ______________________________

Instrukcja:

Proszę zaznaczyć jak często spożywała Pani wskazany produkt **w ciągu ostatniego roku** poprzez **zaznaczenie właściwej kratki** ☒ oraz **wpisanie jednorazowo spożywanej porcji.**

Przykład:

Kwestionariusz częstotliwości spożycia żywności FFQ

Produkty	Miesięcznie <1	1-3	Tygodniowo 1-2	3-4	5-6	Dziennie 1	2	3+	**Ilość** (g/ml) **lub wielkość porcji** (liczba sztuk, kawałków, łyżeczka itp.)
**Produkty zbożowe**									
Pieczywo białe, w tym bułki	☐	☒	☐	☐	☐	☐	☐	☐	1 kajzerka
Pieczywo pełnoziarniste/razowe	☐	☐	☐	☐	☐	☐	☒	☐	1 gruba kromka
Pieczywo chrupkie/wafle ryżowe	☒	☐	☐	☐	☐	☐	☐	☐	____________
Makaron jasny	☐	☒	☐	☐	☐	☐	☐	☐	100 g ugotowanego
Makaron razowy	☐	☐	☒	☐	☐	☐	☐	☐	1 szklanka suchego
Ryż biały	☐	☐	☐	☒	☐	☐	☐	☐	4 łyżki ugotowanego

Produkty	Miesięcznie <1	Miesięcznie 1-3	Tygodniowo 1-2	Tygodniowo 3-4	Tygodniowo 5-6	Dziennie 1	Dziennie 2	Dziennie 3+	**Ilość** (g/ml) **lub wielkość porcji** (liczba sztuk, kawałków, łyżeczka itp.)
**Produkty zbożowe**									
Pieczywo białe, w tym bułki	☐	☐	☐	☐	☐	☐	☐	☐	________
Pieczywo pełnoziarniste/razowe	☐	☐	☐	☐	☐	☐	☐	☐	________
Pieczywo chrupkie/wafle ryżowe	☐	☐	☐	☐	☐	☐	☐	☐	________
Makaron jasny	☐	☐	☐	☐	☐	☐	☐	☐	________
Makaron razowy	☐	☐	☐	☐	☐	☐	☐	☐	________
Ryż biały	☐	☐	☐	☐	☐	☐	☐	☐	________
Ryż brązowy	☐	☐	☐	☐	☐	☐	☐	☐	________
Kasze drobne (manna, mazurska, kuskus)	☐	☐	☐	☐	☐	☐	☐	☐	________
Kasze grube (gryczana, pęczak, jaglana)	☐	☐	☐	☐	☐	☐	☐	☐	________
Płatki śniadaniowe słodzone	☐	☐	☐	☐	☐	☐	☐	☐	________
Płatki zbożowe naturalne/musli	☐	☐	☐	☐	☐	☐	☐	☐	________
Otręby pszenne/żytnie/owsiane	☐	☐	☐	☐	☐	☐	☐	☐	________
**Produkty mleczne**									
Mleko odtłuszczone *(0-0,5% tł)*	☐	☐	☐	☐	☐	☐	☐	☐	________
Mleko półtłuste *(1,5-2% tł)*	☐	☐	☐	☐	☐	☐	☐	☐	________
Mleko pełne (3,2-3,5% tł)	☐	☐	☐	☐	☐	☐	☐	☐	________
Kefir/jogurt (0% tł)	☐	☐	☐	☐	☐	☐	☐	☐	________
Kefir/jogurt (1,5-2% tł)	☐	☐	☐	☐	☐	☐	☐	☐	________
Twaróg tłusty	☐	☐	☐	☐	☐	☐	☐	☐	________
Twaróg chudy/ półtłusty	☐	☐	☐	☐	☐	☐	☐	☐	________
Ser topiony	☐	☐	☐	☐	☐	☐	☐	☐	________
Ser żółty	☐	☐	☐	☐	☐	☐	☐	☐	________
Ser pleśniowy	☐	☐	☐	☐	☐	☐	☐	☐	________
Ser sałatkowy (feta, mozzarella)	☐	☐	☐	☐	☐	☐	☐	☐	________
Napoje roślinne	☐	☐	☐	☐	☐	☐	☐	☐	________
**Mięso i produkty mięsne**									
Wieprzowina, w tym mielona	☐	☐	☐	☐	☐	☐	☐	☐	________
Wołowina/cielęcina, w tym mielona	☐	☐	☐	☐	☐	☐	☐	☐	________
Kurczak/indyk, w tym mielony	☐	☐	☐	☐	☐	☐	☐	☐	________
Wątróbka/podroby	☐	☐	☐	☐	☐	☐	☐	☐	________
Drobiowa szynka/polędwica	☐	☐	☐	☐	☐	☐	☐	☐	________
Wieprzowa szynka/polędwica	☐	☐	☐	☐	☐	☐	☐	☐	________

Kwestionariusz częstotliwości spożycia żywności (FFQ-NutriForHer)

**Produkty**	**Miesięcznie**		**Tygodniowo**			**Dziennie**			**Ilość** (g/ml) **lub wielkość porcji** (liczba sztuk, kawałków, łyżeczka itp.)
	**<1**	**1-3**	**1-2**	**3-4**	**5-6**	**1**	**2**	**3+**	
Kiełbasa	☐	☐	☐	☐	☐	☐	☐	☐	________________
Parówki	☐	☐	☐	☐	☐	☐	☐	☐	________________
Salami/boczek/mielonka	☐	☐	☐	☐	☐	☐	☐	☐	________________
Pasztet	☐	☐	☐	☐	☐	☐	☐	☐	________________
**Jaja, ryby i owoce morza**									
Jaja	☐	☐	☐	☐	☐	☐	☐	☐	________________
Śledź	☐	☐	☐	☐	☐	☐	☐	☐	________________
Makrela	☐	☐	☐	☐	☐	☐	☐	☐	________________
Łosoś	☐	☐	☐	☐	☐	☐	☐	☐	________________
Tuńczyk	☐	☐	☐	☐	☐	☐	☐	☐	________________
Sardynki	☐	☐	☐	☐	☐	☐	☐	☐	________________
Dorsz	☐	☐	☐	☐	☐	☐	☐	☐	________________
Mintaj	☐	☐	☐	☐	☐	☐	☐	☐	________________
Inne ryby: ___________________	☐	☐	☐	☐	☐	☐	☐	☐	________________
Krewetki	☐	☐	☐	☐	☐	☐	☐	☐	________________
Inne owoce morza: ___________	☐	☐	☐	☐	☐	☐	☐	☐	________________
**Warzywa**									
Sałata	☐	☐	☐	☐	☐	☐	☐	☐	________________
Szpinak	☐	☐	☐	☐	☐	☐	☐	☐	________________
Rukola/roszponka	☐	☐	☐	☐	☐	☐	☐	☐	________________
Jarmuż	☐	☐	☐	☐	☐	☐	☐	☐	________________
Ziemniaki	☐	☐	☐	☐	☐	☐	☐	☐	________________
Marchew	☐	☐	☐	☐	☐	☐	☐	☐	________________
Seler/pietruszka	☐	☐	☐	☐	☐	☐	☐	☐	________________
Cebula/por	☐	☐	☐	☐	☐	☐	☐	☐	________________
Buraki	☐	☐	☐	☐	☐	☐	☐	☐	________________
Kalafior	☐	☐	☐	☐	☐	☐	☐	☐	________________
Brokuły	☐	☐	☐	☐	☐	☐	☐	☐	________________
Kapusta (biała/czerwona/brukselka)	☐	☐	☐	☐	☐	☐	☐	☐	________________
Kiszonki (kapusta/ogórki)	☐	☐	☐	☐	☐	☐	☐	☐	________________
Papryka	☐	☐	☐	☐	☐	☐	☐	☐	________________
Pomidor	☐	☐	☐	☐	☐	☐	☐	☐	________________
Fasolka szparagowa	☐	☐	☐	☐	☐	☐	☐	☐	________________
Groszek zielony	☐	☐	☐	☐	☐	☐	☐	☐	________________

Produkty	Miesięcznie <1	Miesięcznie 1-3	Tygodniowo 1-2	Tygodniowo 3-4	Tygodniowo 5-6	Dziennie 1	Dziennie 2	Dziennie 3+	**Ilość** (g/ml) **lub wielkość porcji** (liczba sztuk, kawałków, łyżeczka itp.)
Dynia	☐	☐	☐	☐	☐	☐	☐	☐	________________
Kukurydza	☐	☐	☐	☐	☐	☐	☐	☐	________________
Oliwki	☐	☐	☐	☐	☐	☐	☐	☐	________________
Mix warzywny mrożony	☐	☐	☐	☐	☐	☐	☐	☐	________________
Inne warzywa_______________	☐	☐	☐	☐	☐	☐	☐	☐	________________
**Owoce**									
Banan	☐	☐	☐	☐	☐	☐	☐	☐	________________
Jabłko/gruszka	☐	☐	☐	☐	☐	☐	☐	☐	________________
Pomarańcza/grejpfrut/mandarynka	☐	☐	☐	☐	☐	☐	☐	☐	________________
Kiwi	☐	☐	☐	☐	☐	☐	☐	☐	________________
Brzoskwinia/morela	☐	☐	☐	☐	☐	☐	☐	☐	________________
Winogrona	☐	☐	☐	☐	☐	☐	☐	☐	________________
Wiśnie/czereśnie	☐	☐	☐	☐	☐	☐	☐	☐	________________
Śliwki	☐	☐	☐	☐	☐	☐	☐	☐	________________
Arbuz/melon	☐	☐	☐	☐	☐	☐	☐	☐	________________
Mango/ananas	☐	☐	☐	☐	☐	☐	☐	☐	________________
Truskawki/maliny	☐	☐	☐	☐	☐	☐	☐	☐	________________
Borówki/jagody/żurawina	☐	☐	☐	☐	☐	☐	☐	☐	________________
Awokado	☐	☐	☐	☐	☐	☐	☐	☐	________________
Suszone owoce (rodzynki/morele itp.)	☐	☐	☐	☐	☐	☐	☐	☐	________________
**Nasiona roślin strączkowych (suche nasiona)**									
Groch	☐	☐	☐	☐	☐	☐	☐	☐	________________
Soczewica	☐	☐	☐	☐	☐	☐	☐	☐	________________
Fasola Jaś/czerwona	☐	☐	☐	☐	☐	☐	☐	☐	________________
Soja/produkty sojowe	☐	☐	☐	☐	☐	☐	☐	☐	________________
Ciecierzyca	☐	☐	☐	☐	☐	☐	☐	☐	________________
**Orzechy i nasiona oleiste**									
Migdały	☐	☐	☐	☐	☐	☐	☐	☐	________________
Orzechy laskowe	☐	☐	☐	☐	☐	☐	☐	☐	________________
Orzechy włoskie	☐	☐	☐	☐	☐	☐	☐	☐	________________
Orzechy arachidowe niesolone	☐	☐	☐	☐	☐	☐	☐	☐	________________
Pistacje	☐	☐	☐	☐	☐	☐	☐	☐	________________
Solone orzechy	☐	☐	☐	☐	☐	☐	☐	☐	________________
Inne orzechy: ________________	☐	☐	☐	☐	☐	☐	☐	☐	________________

Kwestionariusz częstotliwości spożycia żywności (FFQ-NutriForHer)

Produkty	Miesięcznie <1	Miesięcznie 1-3	Tygodniowo 1-2	Tygodniowo 3-4	Tygodniowo 5-6	Dziennie 1	Dziennie 2	Dziennie 3+	**Ilość** (g/ml) **lub wielkość porcji** (liczba sztuk, kawałków, łyżeczka itp.)
Mieszanka studencka	☐	☐	☐	☐	☐	☐	☐	☐	________________
Masło orzechowe	☐	☐	☐	☐	☐	☐	☐	☐	________________
Pestki słonecznika	☐	☐	☐	☐	☐	☐	☐	☐	________________
Pestki dyni	☐	☐	☐	☐	☐	☐	☐	☐	________________
Nasiona lnu	☐	☐	☐	☐	☐	☐	☐	☐	________________
**Cukier i wyroby cukiernicze**									
Cukier	☐	☐	☐	☐	☐	☐	☐	☐	________________
Miód	☐	☐	☐	☐	☐	☐	☐	☐	________________
Dżem/marmolada	☐	☐	☐	☐	☐	☐	☐	☐	________________
Słodzik: ____________________	☐	☐	☐	☐	☐	☐	☐	☐	________________
Pieczywo cukiernicze	☐	☐	☐	☐	☐	☐	☐	☐	________________
Ciastka francuskie	☐	☐	☐	☐	☐	☐	☐	☐	________________
Ciastka/herbatniki	☐	☐	☐	☐	☐	☐	☐	☐	________________
Czekolada mleczna	☐	☐	☐	☐	☐	☐	☐	☐	________________
Czekolada gorzka	☐	☐	☐	☐	☐	☐	☐	☐	________________
Czekoladowe batony/wafelki/cukierki	☐	☐	☐	☐	☐	☐	☐	☐	________________
Cukierki/krówki	☐	☐	☐	☐	☐	☐	☐	☐	________________
Lody/sorbety	☐	☐	☐	☐	☐	☐	☐	☐	________________
Nutella/kremy czekoladowe	☐	☐	☐	☐	☐	☐	☐	☐	________________
**Napoje bezalkoholowe**									
Woda mineralna/źródlana	☐	☐	☐	☐	☐	☐	☐	☐	________________
Herbata (czarna, zielona)	☐	☐	☐	☐	☐	☐	☐	☐	________________
Kawa	☐	☐	☐	☐	☐	☐	☐	☐	________________
Soki owocowe (pomarańczowy/jabłkowy)	☐	☐	☐	☐	☐	☐	☐	☐	________________
Soki warzywne (marchwiowy/pomidorowy)	☐	☐	☐	☐	☐	☐	☐	☐	________________
Inne soki____________________	☐	☐	☐	☐	☐	☐	☐	☐	________________
Napoje owocowe/kompot	☐	☐	☐	☐	☐	☐	☐	☐	________________
Napoje typu cola	☐	☐	☐	☐	☐	☐	☐	☐	________________
Napoje izotoniczne	☐	☐	☐	☐	☐	☐	☐	☐	________________
Napoje energetyzujące	☐	☐	☐	☐	☐	☐	☐	☐	________________
**Napoje alkoholowe**									
Piwo	☐	☐	☐	☐	☐	☐	☐	☐	________________
Wino białe/szampan	☐	☐	☐	☐	☐	☐	☐	☐	________________
Wino czerwone	☐	☐	☐	☐	☐	☐	☐	☐	________________

Kwestionariusz częstotliwości spożycia żywności (FFQ-NutriForHer)

**Produkty**	**Miesięcznie** <1	1-3	**Tygodniowo** 1-2	3-4	5-6	**Dziennie** 1	2	3+	**Ilość** (g/ml) **lub wielkość porcji** (liczba sztuk, kawałków, łyżeczka itp.)
Drinki na bazie alkoholu	☐	☐	☐	☐	☐	☐	☐	☐	________________
Wódka/mocne alkohole	☐	☐	☐	☐	☐	☐	☐	☐	________________
Inne alkohole:_______________	☐	☐	☐	☐	☐	☐	☐	☐	________________
**Tłuszcze i produkty do smarowania pieczywa**									
Oliwa z oliwek	☐	☐	☐	☐	☐	☐	☐	☐	________________
Olej słonecznikowy	☐	☐	☐	☐	☐	☐	☐	☐	________________
Olej rzepakowy	☐	☐	☐	☐	☐	☐	☐	☐	________________
Olej lniany	☐	☐	☐	☐	☐	☐	☐	☐	________________
Olej kokosowy	☐	☐	☐	☐	☐	☐	☐	☐	________________
Masło (82% tłuszczu)	☐	☐	☐	☐	☐	☐	☐	☐	________________
Śmietana (9-18% tłuszczu)	☐	☐	☐	☐	☐	☐	☐	☐	________________
Śmietanka (30-36% tłuszczu)	☐	☐	☐	☐	☐	☐	☐	☐	________________
Margaryna: ____________________	☐	☐	☐	☐	☐	☐	☐	☐	________________
Masmix: ______________________	☐	☐	☐	☐	☐	☐	☐	☐	________________
Smalec	☐	☐	☐	☐	☐	☐	☐	☐	________________
Inne: _________________________	☐	☐	☐	☐	☐	☐	☐	☐	________________
**Inne produkty**									
Chipsy/frytki	☐	☐	☐	☐	☐	☐	☐	☐	________________
Popcorn	☐	☐	☐	☐	☐	☐	☐	☐	________________
Pizza/hamburger/kebab	☐	☐	☐	☐	☐	☐	☐	☐	________________
Naleśniki/pierogi	☐	☐	☐	☐	☐	☐	☐	☐	________________
Majonez	☐	☐	☐	☐	☐	☐	☐	☐	________________
Ketchup	☐	☐	☐	☐	☐	☐	☐	☐	________________
